# An enhanced domestication method for uncultured bacteria

**DOI:** 10.1093/ismeco/ycag062

**Published:** 2026-03-16

**Authors:** Andrew G Morrison, Raphaella Jackson, Paul S Freemont, Harry H Low

**Affiliations:** Department of Infectious Disease, Imperial College, London, SW7 2AZ, United Kingdom; Department of Infectious Disease, Imperial College, London, SW7 2AZ, United Kingdom; Department of Infectious Disease, Imperial College, London, SW7 2AZ, United Kingdom; Department of Infectious Disease, Imperial College, London, SW7 2AZ, United Kingdom

**Keywords:** bacterial enhanced domestication (EDEN), uncultured bacteria, microbial cultivation, 16S rDNA amplicon sequencing, antimicrobial discovery, 3D-printed microwell plate

## Abstract

When environmental bacteria transition to laboratory conditions, a process termed domestication, the shift from the native habitat to a culture medium often reduces cell cultivability. Consequently, most bacteria remain uncultured using standard techniques, leaving the majority of their diversity unexplored. Here we introduce an enhanced domestication (EDEN) method for bacterial cultivation, which acclimatises environmental bacteria to culture media through a controlled and gradual exposure. To facilitate EDEN, we develop a 3D-printable microwell plate incorporating growth chambers integrated with a continuous-flow media reservoir. Using amplicon sequencing, we show that EDEN-acclimatised bacterial polycultures grow as distinct populations with significantly greater diversity and likely-uncultured taxa compared with standard cultivation methods. Similarly, EDEN-acclimatised bacterial monocultures show three-fold greater diversity and tenfold more likely-uncultured taxa. EDEN also doubled the cultivability of agarose-encapsulated microcolonies. Finally, we demonstrate the utility of EDEN by isolating a previously uncultured bacterium exhibiting broad-spectrum antimicrobial activity against drug-resistant pathogens.

## Introduction

Culturing microorganisms is essential for many aspects of fundamental research, biotechnology, and drug discovery. However, conventional cultivation techniques are inefficient with 99% of microbes remaining uncultured [1, 2]. Certain bacterial groups, like those in the phyla Acidobacteriota, Verrucomicrobiota and Gemmatimonadota, are particularly difficult to cultivate in the laboratory [3–5]. Such poorly cultured microbes, as well as many others, harbour unexplored biosynthetic gene clusters (BGCs) often coding for natural products with uncharted chemical structure important for drug development [6–8]. Consequently, developing methods to access uncultured bacteria will promote the discovery of novel therapeutics including antibiotics, antifungals and antitumorals [9–12].

Typically, standard laboratory cultivation incubates bacteria with a culture media mixed in liquid or a hydrogel support like agar [13]. This conventional approach may be optimised by varying nutrient composition [14], incubation temperature and duration, regulating atmosphere, or providing suitable electron acceptors [15]. However, many additional innovative approaches have been devised for enhancing the biodiversity, novelty and yield of environmental microbial cultivations [5]. These include exploiting symbiotic co-cultivation to provide critical growth factors [16, 17] or signalling compounds present in native habitats [18–21]. Low-nutrient media derived from native habitats are effective at cultivating previously uncultured microorganisms by mimicking their natural conditions [22–25]. A powerful variant of this approach uses membrane-diffusion-chamber technology [26, 27], which inspired the isolation chip (iChip) for the *in situ* cultivation of isolated microorganisms within their native environment [28]. Alternative diffusion-based methods include a hollow-fibre membrane chamber device [29], a diffusion bioreactor [30], and a soil substrate membrane system [31].

Though techniques such as *in situ* cultivation improve the likelihood of microbial domestication, the dependency on the native environment for cultivation is affected by environmental factors including seasonal variation [22, 30]. Moreover, both *in situ* and standard cultivation methods often expose microbes to abrupt transitions from their native habitat to culture media as a necessary step towards laboratory domestication [19, 27, 28, 32–35]. Such transitions may trigger a shock response that can arrest growth due to factors including osmotic stress [36], reactive oxygen species [37], or nutrient shift [38, 39]. These abrupt changes can also reduce the viability of oligotrophs adapted to nutrient-poor environments [40–42]. However, microbial growth in relatively nutrient-rich culture media may be achieved, at least for some oligotrophs, if they are given sufficient time for adaptation [43] or exposed to appropriate conditioning steps [44]. Similarly, microbial diversity can be enhanced by filtering out fast-growing species and acclimatising the remaining community from low- to high-nutrient culture media using fed-batch transitions [45]. In both fed-batch and batch cultures, microbial growth dynamics and diversity may be influenced by the accumulation of metabolic waste, toxins and oxidative stress [46–48], microbe-induced changes in pH [49], and nutrient depletion upon prolonged incubation time. This contrasts with non-limited natural habitats where continuous flow may replenish low-nutrient levels and remove waste products detrimental to growth. In this way, continuous flow chemostat-like systems have proven effective in growing deep marine sediment archaea [49].

In this study, we develop an enhanced domestication (EDEN) cultivation method that transitions microbes from native media to culture media via a gradual and tightly controlled acclimatisation process. Native media refers to a growth medium derived directly from the natural habitat in which the microbes are sourced. The approach minimises microbial exposure to abrupt media and environmental transitions compared with standard cultivation, thereby limiting potential nutrient shock effects. This is achieved using a 3D-printed EDEN microwell plate that incorporates an exchangeable continuous-flow media reservoir supporting metabolic waste removal, and control over the nutrient environment in which the microbes grow. The continuous flow within the EDEN microwell plate aims to better mimic some natural habitats compared with batch and fed-batch methods, whilst supporting high-throughput microbial cultivation. The effectiveness of EDEN to cultivate bacteria over standard cultivation methods is established by assessing the diversity, abundance and structure of bacterial polycultures and monocultures, as well as the cultivability of agarose-encapsulated microcolonies. Finally, we demonstrate the utility of EDEN by cultivating a novel bacterial species with potent antimicrobial properties.

## Materials and methods

### Cells and media

Unless specified, all reagents were purchased from Sigma-Aldrich, UK. Reasoner’s 2A (R2A) media [50] was prepared using 0.5 g yeast extract, 0.5 g proteose peptone, 0.5 g casamino acids, 0.5 g glucose, 0.5 g soluble starch, 0.3 g dipotassium phosphate, 0.05 g magnesium sulfate heptahydrate, 0.3 g of sodium pyruvate ±15 g bacteriological agar/litre (L). TY agar was prepared according to DSMZ Medium1143 using 5.0 g tryptone, 3.0 g yeast extract, 0.9 g calcium chloride dihydrate and 10 g bacteriological agar/L. Mueller Hinton (MH) agar contained 2.0 g beef heart infusion, 17.5 g casein hydrolysate, 1.5 g starch and 15 g bacteriological agar/L. Pondwater (pH 6.5) was collected with sediment 10 cm below the water surface from King’s Mere, Putney Heath, UK (51° 26′ 42.7194″ N, 0° 13′ 48.1908″ W) in 20 L high-density polyethylene buckets throughout March–June 2023. Two independent collections two weeks apart were used for all experiments and independent replicates. Pondwater was processed on the day of collection to generate sterile-filtered pondwater (SFPW) media used for inoculum preparation, and coarse-filtered pondwater (CFPW) media delivered to EDEN reservoirs, with both stored at room temperature. SFPW media was prepared by centrifuging pondwater at 4,000 g for 10 min followed by vacuum filtration using a 0.1 μm polyether sulfone filter (ThermoFisher). CFPW was prepared by filtering pondwater through a 1 mm stainless steel sieve to remove larger sediment particles whilst retaining finer particles and native microflora. *Bacillus subtilis* 168 was kindly donated by Josef Altenbuchner, University of Stuttgart, Germany. *Pseudomonas putida* KT2440 (ATCC No. 47054) and *Paenibacillus polymyxa* 25A2 (ATCC 842) were purchased from ATCC. Clinical isolates of MRSA, MSSA, vancomycin-resistant *Enterococcus faecium* (VRE) or *Escherichia coli* extended-spectrum beta-lactamase (ESBL) were kindly donated by Shiranee Sriskandan, Colebrook Laboratory, NIHR Imperial Biomedical Research Centre, UK.

### EDEN plate fabrication and validation

EDEN plate models were created using AutoCAD 2022 (Autodesk) and exported as standard tessellation language (STL) files for digital slicing with Ultimaker-Cura (v5.1.0). The Gcode was then uploaded to a Prusa i3 MK3S+ 3D printer, utilising natural PLA thermoplastic (Polymaker) on a 60°C heated polyetherimide build plate. Extrusion was maintained at 220°C nozzle temperature, 100 μm layer height, 100% infill density, 105% flow rate, and a 60 mm/s printing speed. Coasting was enabled using default parameters. After printing the lower reservoir, a post-processing pause script was initiated with printer head parking positioned at X = 5 mm and Y = 195 mm. A semi-permeable polycarbonate membrane with 0.05 μm pore (Sterlitech, SKU: PCT0053001) was then overlaid over the reservoir and secured to the printer bed with PVA glue. Printing was continued with the first membrane layer printing at 6 mm/s before continuing at 60 mm/s. EDEN plate inlet and outlet ports that connected to peristaltic pump tubing had support materials removed, before being polished with P100 sandpaper and wrapped with parafilm for a water-tight connection. EDEN plates were sterilised by rinsing with distilled water, followed by overnight immersion in 70% ethanol. The plates were washed with sterile distilled water and air-dried in a biosafety cabinet. Reservoirs were flushed with sterile distilled water before use. For all experiments, 96- or 384-well EDEN plates were filled with 50 μl or 20 μl per well, respectively.

To validate the biocompatibility of the EDEN plate, *B. subtilis* 168 was cultivated for 24 h at 28°C in 10 ml R2A broth with 220 rpm shaking. A 1% low-gelling-point R2A agarose solution was prepared by dissolving agarose powder in sterile R2A medium via boiling, followed by sterile filtration of the molten mixture through a 0.2 μm PES syringe filter. The molten agarose was cooled to 37°C, at which point it remained liquid, before the addition and dilution of *B. subtilis* culture to yield approximately one cell per well in 384-well EDEN plates.

The agarose-cell suspension was dispensed into test EDEN plates printed on a semi-permeable polycarbonate membrane (0.05 μm pore size) lacking lower reservoirs, in 20 μL volumes. Plates were sealed with clear polyester sealing film and centrifuged at 500 rpm for 2 s to ensure contact between the liquid mixture and the membrane well floor. The EDEN plates were incubated at room temperature to allow solidification of the low-gelling-point agarose containing encapsulated bacteria, after which they were placed on filter paper moistened with phosphate-buffered saline and cultivated at 26°C for 14 days. Parallel cultivations were compared using standard clear 384-well plates (Greiner Bio-One). The number of wells with cultivated microcolonies was then quantified by light microscopy. To validate the integrity of the PC membrane, sterile R2A broth was dispensed into 96- or 384-well EDEN plate growth chambers whilst overnight R2A culture of *P. putida* was injected into the media reservoir. After 14 days of incubation at 26°C, 5 μl of each well from independent EDEN plates (n = 3) was dispensed onto a 1.5% R2A agar plate and cultivated for 48 h at 26°C before quantification of sterile wells.

### Peristaltic pump and media delivery system validation

A custom-built media delivery system was developed to supply up to nine EDEN plates simultaneously although this could be readily scaled. The setup utilised two Arduino Uno microcontrollers: the first controlled three stepper motor–driven peristaltic pumps (Yanmis) via DRV8825 stepper drivers (Youmile). Two of these pumps independently drew sterile R2A media or CFPW media from 2 L containers into a 500 ml Duran bottle with a magnetic stir bar, which constituted a mixing vessel for Acclimatised_EDEN_ plates. The third pump supplied R2A to a separate 500 ml Duran bottle for R2A_EDEN_ plates. The Duran bottles also served as traps to prevent contamination of the media stocks. The second Arduino controlled nine pumps, each delivering media from a Duran bottle to an EDEN plate. For pondwater_EDEN_ samples, CFPW media was pumped directly into the EDEN plates. All pumps were connected to their respective Arduino boards via digital step and direction pins and powered by a 12 V external supply. Media was transported through 1.5 mm inner-diameter silicone tubing (VWR) directly into the EDEN reservoirs. Delivery cycles were automated to occur every 4 h, with each EDEN plate receiving 10 ml of media per cycle—approximately one full reservoir volume at a flow rate of 2.5 ml/min.

To validate the accuracy of the pump system, 96-well EDEN plates (n = 3) were acclimatised to 5 μM of fluorescein sodium salt in 1 mM Tris–HCl pH 8 over an exponential gradient from 0.1% to 100% dye concentration. At each timepoint, the dye concentration was quantified by fluorescent intensity at 515 ± 20 nm (excitation 470 ± 15 nm) on a CLARIOstar plate reader (BMG Labtech). Note that although both the growth chambers and the reservoir closely follow the theoretical exponential gradient (Supplementary Fig. 2C), modest local variability in shear stress, diffusion rates, and residence time distributions within EDEN reservoirs and growth chambers cannot be excluded. While these potential confounding factors are expected to be comparable across EDEN plates and thus to affect all plates similarly, some contribution to the observed data, however minor, cannot be ruled out.

### Polyculture setup and cultivation

For environmental cell extraction, 1 ml samples of pondwater-sediment slurry, collected from the bed surface where the native pondwater was also sourced, were mixed on the day of collection in 9 ml of SFPW and vortexed with 3 g of 1 mm glass beads for 15 min. The homogenised samples were filtered through a 20 μm filter (pluriSelect) and stained with 5 μM carboxyfluorescein diacetate-succinimidyl ester (CFDA-SE, ThermoFisher) in R2A for 30 min at 23°C. CFDA-SE is a cell-permeable reagent that selectively stains live cells. Fluorescent cell abundances were quantified using a haemocytometer as CFDA-SE positive cells. To initiate polyculture experiments in 96-well EDEN plates, 1.00x10^4^ viable cells suspended in 50 μl of SFPW supplemented with 0.1% R2A were dispensed to each well. Plates were sealed with clear polyester self-adhesive film (Starlab). Cells were sourced from bed surface sediment as a slurry rather than the pondwater fraction due to relatively low cell number in the latter, which would have led to an undesired concentration step during polyculture inoculations. For Acclimatised_EDEN_ experiments, cells were transitioned from native pondwater to R2A over 28 days with exponential increments (to the power of 1.00017134 per minute). In parallel, EDEN plates were setup for R2A_EDEN_ or Pondwater_EDEN_ experiments with cells initially mixed with R2A or SFPW for inoculation, respectively. The same media were then supplied in the continuous flow reservoir following the equivalent flow rate as for Acclimatised_EDEN_. In addition, equivalent cells mixed with R2A were dispensed to 1.5% R2A agar petri dishes for standard cultivation (R2A_standard_). For each cultivation condition, 5 independent EDEN plate replicates were run. Parallel EDEN plates (n = 3) were incubated without cells as sterile controls with growth chambers carefully examined for contamination at the end of each 28 day incubation using both 16S rDNA PCR (as described below) and plating onto R2A agar for a further 28 days incubation at room temperature. All sterile media stocks used for the duration of these experiments, including SFPW and R2A, were similarly examined for contamination. In all cases EDEN growth chambers or stock media remained sterile. Cultivations were conducted on benchtops at room-temperature (23°C). After incubation, EDEN plate wells were harvested by adding 50 μl R2A to each well with resuspension before aspiration of well contents (~100 μl). Colonies from R2A agar plates were collected by resuspending in 1 ml R2A by scraping using an L-spreader. Samples were stored at −20°C and thawed before DNA extraction using an environmental DNA extraction kit (MP Biomedicals). 500 μl of the 20 μm-filtered raw pondwater-sediment slurry (termed raw pondwater as found in Supplementary Fig. 4) was sequenced in parallel for comparison. To estimate viable cell counts, CFDA-SE was added to three randomly selected wells from each EDEN plate replicate. Fluorescent cell counts were quantified using a haemocytometer and averaged across the three random wells.

### Liquid monoculture setup and cultivation

The protocol was the same as for polycultures except for the following changes. 384-well EDEN plates were inoculated with ~1 cell per well (Poisson λ = 1) mixed in 20 μl of desired media according to the specific cultivation condition. To ensure consistent contact of the sample with the membrane after dispensing and to displace air pockets, the plate was centrifuged at 500 rpm for 2 s. For standard R2A cultivation (R2A_standard_), equivalent cells were diluted into R2A with 20 μl dispensed per well in commercially available clear 384 microwell plates sealed with clear polyester self-adhesive film. Parallel EDEN plates (n = 3) were incubated without cells as sterile controls with growth chambers carefully examined for contamination as previously described. Sterile media stocks were similarly examined for contamination using the same procedure.

### Monoculture with agarose encapsulated cells

The protocol was the same as for liquid monocultures except for the following changes. Desired media specific to each cultivation condition was boiled with 1% low-gelling-point agarose, syringe-filtered (0.2 μm) and inoculated with bacteria after cooling to 37°C. 20 μl of cell suspension was pipetted into each well followed by centrifugation at 500 rpm for 2 s and solidification of the agarose at room temperature. For each cultivation condition, 3 independent EDEN plate replicates were run. Parallel EDEN plates (n = 3) were incubated without cells as sterile controls with growth chambers carefully examined for contamination as previously described. Sterile media stocks were similarly examined for contamination using the same procedure. After cultivation, the EDEN plate growth chamber layer with membrane attached was physically wedged apart from the lower reservoir layer. This enabled wells within the growth chamber layer to be readily visualised by light microscopy with grown microcolonies >100 μm counted. Parallel cultivation comparisons of bacteria in 0.1% R2A SFPW ±0.1% R2A were undertaken in commercially available clear 384 microwell plates only.

### Polyculture next-generation sequencing and data analysis

Library preparation and sequencing were performed using an Illumina MiSeq platform with a 2 × 300 bp paired-end read module by Eurofins Genomics (Germany). Libraries were generated by a two-step PCR with first amplification of the bacterial 16S rDNA V3-V4 region (forward: 5-′ TACGGGAGGCAGCAG −3′ and reverse: 5-′ CCAGGGTATCTAATCC -3′) followed by appending of amplicons with sample barcodes [51, 52]. Raw sequences were processed using the Ampliseq pipeline available from nf-core (revision 2.4.0) with default paramaters [53]. This pipeline included read quality analysis with FastQC (v 0.11.9) [54], read trimming using Cutadapt basic (v 3.4) [54], and denoising into amplicon sequence variants (ASVs) using DADA2 (v 1.22.0) [55]. ASVs were assigned taxonomic labels using DADA2 based on the Silva SSU database, release 138. ASVs annotated as mitochondria or chloroplasts were removed, and ASV tables with taxonomic assignments were further analysed in R.

For the non-metric multidimensional scaling (NMDS) plot and permutational multivariate analysis of variance (PERMANOVA), the data was transformed to account for noise and variation in sampling depth. The ASV counts were transformed into relative abundance proportions within each sample and those which accounted for <0.5% of reads within a sample were filtered out to control for spurious ASVs. Subsequently, a Bray–Curtis dissimilarity matrix was computed on the transformed data. For the NMDS plot, the metaMDS function was called with iterative optimisation for a stable solution. Permutational multivariate analysis of variance (PERMANOVA) was conducted using the adonis2 function from the vegan package (v 2.4.3) to assess the significance of sample type in shaping community composition [56]. Venn diagrams were generated using Venny (https://bioinfogp.cnb.csic.es/tools/venny/). Statistical analysis was performed in R and GraphPad Prism 10. For reporting of ANOVA testing, the p value was reported followed by the F statistic with degrees of freedom of the numerator and denominator in parenthesis.

### Liquid monoculture 16S rDNA amplification, sequencing and analysis

After bacterial cultivation in 384 microwell plates or EDEN plates, 10 μl of culture was sampled from each well and lysed in 40 μl of 10 mM Tris–HCl pH 8, 1 mM EDTA, 1% Triton X-100 buffer by heating at 99°C for 12 min. Lysates were placed on ice before 16S rDNA amplification with 27F (5-′ AGA GTT TGA TYM TGG CTC AG −3′) and 907R (5-′ CCG TCA ATT CMT TTG AGT TT −3′) primers [57]. 1 μl lysate volume was added as template to each 20 μl PCR reaction which was run with the following parameters: pre-denaturation at 98°C for 2 min followed by 35 cycles of denaturation at 98°C for 30 s, annealing at 54°C for 40 s, elongation at 72°C for 40 s, and a final single elongation step at 72°C for 5 min. An equal fraction of PCR product from each cultivation condition was subsampled for Sanger sequencing. Using Geneious Prime (Biomatters), sequences were quality-checked (Q30) and length filtered (>300 bp), before manual inspection. Using Geneious Assembler under default parameters, sequences were then grouped into exact genetic variants (Sanger sequencing variants, SSVs) by assembling them at 100% identity, so that identical sequences were grouped together. Each group of identical sequences formed a consensus or contig representing one SSV. Any read that did not match others at 100% identity was kept as separate SSVs (a singleton). This ensured every unique sequence, whether grouped or ungrouped, was counted as a distinct SSV. Distinct SSVs were exported as FASTA files and classified using SILVA Incremental Aligner (SINA v1.2.12) with a minimum query sequence identity threshold of 95%, corresponding to genus-level taxonomic assignment [58].

### Antimicrobial detection by agar overlay

For initial screening of antimicrobial activity, isolates subcultivated from Acclimatised_EDEN_ plates were transferred to the centre of a new R2A agar petri dish and cultivated for 14 days at 23°C. MRSA, used as reporter cells, were cultivated in 10 ml MH broth overnight at 30°C before 1/500 dilution into MH 1% agar maintained at 40°C as a liquid. The cell-agar suspension was then mixed and dispensed on top of the isolate colonies and allowed to solidify before overnight incubation at 30°C. Antimicrobial activity was detected by MRSA growth inhibition clearance zones surrounding the isolate colonies.

### Culture and extract preparation of Isolate-95

Isolate-95 exhibiting antimicrobial activity was cultivated for 48 h at 28°C in 10 ml R2A broth with 220 rpm shaking. 100 μl of ~1.00x10^8^ cells per ml culture was spread each on 15 square bioassay dishes (245 × 245 × 25 mm, Thermo Scientific) containing 200 ml of TY agar. Plates were incubated for 6 days at 28°C before pulverisation of agar and addition to 3 L ethyl acetate in two 2 L Duran bottles. Extraction was left overnight at 8°C with shaking at 160 rpm. The extract was then filtered from the agar using a 0.5 mm sieve and decanted before evaporation at 30°C in a rotary evaporator (BUCHI). Dried extracts were reconstituted at 2000× concentration in 50% methanol:water and filtered at 0.2 μm to remove debris. Sterile media extract was prepared in parallel using the same protocol except without added bacterial inoculum.

### Bacteria growth inhibition assay

Bacterial pathogens ESBL *E. coli*, vancomycin-resistant *Enterococcus faecium*, MRSA, MSSA, *B. subtilis* and *Pseudomonas putida* were cultivated overnight in 10 ml MH broth with shaking at 220 rpm and 37°C. ~1.00 × 10^5^ cells per ml were inoculated into clear-bottom 96 well plates (Corning) in 75 μl MH broth containing 7.5 μl of either Isolate-95 fermentation extract, sterile media extract or MH broth only. Lids were parafilm-sealed and incubated for 21 h at 37°C and 500 rpm. Throughout this time absorbance was measured at OD600 to assess bacterial growth on a CLARIOstar plate reader (BMG Labtech). Data was normalised to a background of OD600 = 0.02.

### Isolate-95 genome sequencing and analysis

Isolate-95 was cultivated in 10 ml R2A for 48 h shaking at 220 rpm and 28°C. The culture was centrifuged at 4000 rpm for 10 min and washed once in PBS before extraction of genomic DNA using an environmental DNA extraction kit (MP Biomedicals). Genomic DNA was sequenced using Oxford Nanopore Technology (Plasmidsaurus). The assembled genome was uploaded to antiSMASH (v 8.0) under relaxed parameters for identification of BGCs. For genome-based taxonomic analysis, whole-genome sequence data were uploaded to the Type (Strain) Genome Server (TYGS; https://tygs.dsmz.de) for genome-based taxonomic analysis [59]. Nomenclatural information, synonymy and associated taxonomic literature were retrieved via TYGS’s sister resource, the List of Prokaryotic names with Standing in Nomenclature (LPSN; https://lpsn.dsmz.de). Closely related type strains were determined using two complementary approaches: (i) comparison of user genomes against all available type strain genomes in the TYGS database using the MASH algorithm to approximate intergenomic relatedness, selecting the 10 strains with the smallest MASH distances per genome [60]; and (ii) extraction of 16S rDNA sequences using RNAmmer [61], followed by BLAST comparison against 16S rDNA sequences of type strains in the TYGS database to identify the top 50 matches by bitscore, from which precise intergenomic distances were calculated using the Genome BLAST Distance Phylogeny (GBDP) method under the “coverage” algorithm and distance formula d5 [62]. For phylogenomic inference, pairwise genome comparisons were conducted using GBDP under the “trimming” algorithm and distance formula d5, with 100 distance replicates calculated per comparison. Digital DNA–DNA hybridization (dDDH) values and confidence intervals were computed using the recommended settings of GGDC 4.0. Intergenomic distances were used to infer a balanced minimum evolution tree with branch support using FASTME 2.1.6.1 including SPR postprocessing [63], with support values derived from 100 pseudo-bootstrap replicates; trees were midpoint-rooted and visualised using iTOL [64]. Type-based species clustering was performed using a 70% dDDH threshold radius around each type strain, and subspecies delineation applied a 79% dDDH threshold as previously introduced [65]. Further analysis was performed using the Microbial Genomes Atlas (MiGA) against the TypeMat reference database to calculate average amino acid identity (AAI) values and estimate rank-specific taxonomic novelty *P*-values [66].

## Results

### EDEN concept and workflow

**Figure 1:**
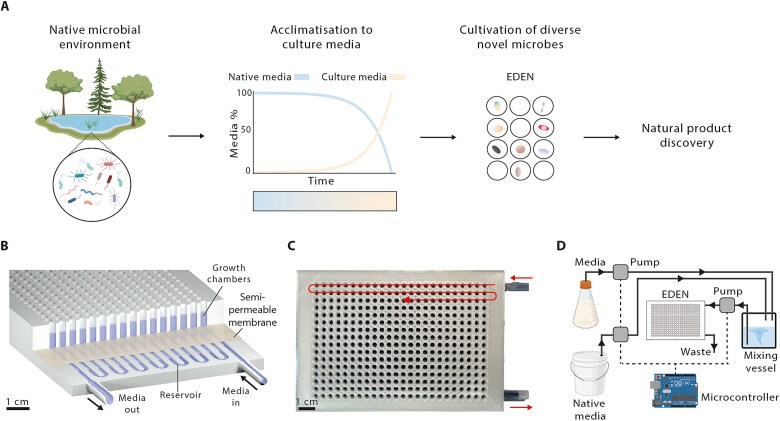
EDEN workflow and plate design. A, conceptual workflow of the EDEN pipeline illustrating the gradual acclimatisation of environmental bacteria to laboratory culture conditions for novel natural product discovery. B, rendered cutaway view of EDEN plate architecture showing the continuous-flow reservoir connected to microbial growth chambers via a semi-permeable membrane. C, top view photograph of a 384-well EDEN plate. Media flow path is overlaid (red arrows). D, schematic of the EDEN experimental setup. Native media and culture media are combined in a mixing vessel and channeled to the EDEN plate via an automated peristaltic pump system directed by a programmable microcontroller. Created partially with BioRender.com.

To enable bacteria to transition from a native media to a culture media via a tightly controlled and gradual acclimatisation process to increase cultivation likelihood and diversity, we developed the EDEN cultivation method (Fig. 1A). Here, a microbial cultivation plate, termed the EDEN plate, was designed with growth chambers compatible with standard 96- or 384-well plate formats, integrated with a continuous flow reservoir (Fig. 1B and C). A semi-permeable polycarbonate membrane separated the growth chambers from the reservoir, preventing bacterial passage whilst permitting nutrient and growth factor diffusion as well as metabolic waste removal. EDEN plates were 3D printed as a single object with the print-pause-print technique integrating the membrane without any requirement for adhesive (Supplementary Fig. 1) [67]. The integrity of the membrane was not compromised during printing and remained impervious to bacteria (Supplementary Fig. 2A). Similarly, the PLA plastic used for printing was biocompatible, as tested with the common environmental bacterium *B. subtilis* (Supplementary Fig. 2B). To control media flow, an automated peristaltic pump and fluidic system was connected to the EDEN plate with native and culture media channels merging in a mixing vessel before entering the reservoir (Figs 1B–D). The proportion of native media to culture media was controlled by adjusting flow rate in each channel, allowing for programmable acclimatisation regimes. We validated the peristaltic pumps by generating an exponential gradient within the EDEN plate, which we tracked by incorporating a fluorescent dye into one of the channels. Across measured timepoints, dye concentrations were within ±3.1% and ± 5.3% of theoretical values in both EDEN plate reservoir and growth wells, respectively (Supplementary Fig. 2C).

As the EDEN plate reservoir operates with liquids or dilute slurries, pondwater-sediment slurry from an urban pond was used as a source of bacteria to seed EDEN plates and to provide native media. R2A was chosen as the target culture media for microbial domestication (52). Acclimatisation was implemented with native media gradually substituted for R2A following an exponential gradient over a 28-day period (Fig. 1A). We evaluated this cultivation strategy using three experimental setups within EDEN plates (Supplementary Fig. 3): a polyculture or monoculture in liquid media inoculated with ~1.0 × 10^4^ or ~1 cell per well, respectively; and a monoculture with ~1 cell per well embedded in agarose.

### Bacterial polycultures acclimatised with EDEN yielded distinct and diversified communities

**Figure 2:**
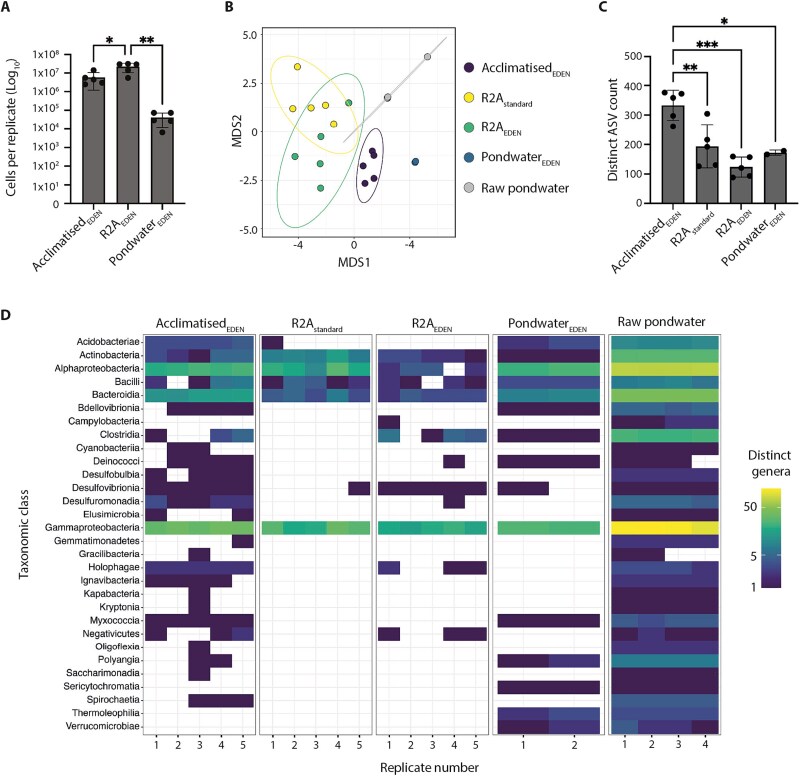
Acclimatised_EDEN_ polycultures yield distinct and diversified bacterial communities. A, mean viable cell counts per replicate for EDEN plate-based cultivation conditions, quantified by cell fluorescent labelling. B, NMDS plot (stress = 0.09) showing community dissimilarity from up to five replicates of different cultivation conditions and the native habitat (raw pondwater-sediment slurry). Ellipses show the clustering of sample groups under a multivariate t-distribution at a 90% confidence interval. C, distinct ASV count per cultivation condition. Replicates are shown as black dots. Bar height indicates the mean ASV count, error bars indicate 1 s standard deviations. D, heatmap showing the relative abundance and distribution of genera within each cultivation condition and raw pondwater-sediment slurry, grouped by taxonomic class. Statistical significance: ^*^P < .05, ^**^P < .01, ^***^P < .001 (one-way ANOVA with Tukey’s multiple comparison test).

We first tested the effects of EDEN on bacterial polycultures. Here, ~1.0 × 10^4^ bacteria extracted from pondwater-sediment slurry were inoculated into the growth chambers of a 96-well EDEN plate for acclimatisation from native pondwater to R2A (Acclimatised_EDEN_). To control for possible confounding variables introduced by the EDEN method, equivalent EDEN plates were run with continually flowing R2A (R2A_EDEN_) or pondwater (Pondwater_EDEN_). For comparison, the same bacterial sample was plate-streaked onto R2A agar petri dishes for standard cultivation and incubation (R2A_standard_). For each of these four cultivation conditions, five independent replicates were set up. After 28 days, viable cells were counted across the three EDEN plate cultivation conditions. Both Acclimatised_EDEN_ and R2A_EDEN_ showed substantial growth, reaching cell densities of 5.9 × 10^6^ ± 4.7 × 10^6^, n = 5 (mean ± s.d., n = independent replicates) and 2.2 × 10^7^ ± 1.0 × 10^6^, n = 5, respectively (ANOVA *P* < .0002 F (2, 12) = 1.999; Fig. 2A). Notably, R2A_EDEN_ yielded approximately four times more cells than Acclimatised_EDEN_ (Tukey HSD: *P* = .0106), likely because continuous culture media replenishment maintained high nutrient availability, supporting greater cell densities. In contrast, Pondwater_EDEN_ showed minimal growth (4.2 × 10^4^ ± 3.0 × 10^4^, n = 5) comparable to the initial inoculum (~1.0 × 10^4^; Fig. 2A) and significantly lower than R2A_EDEN_ (Tukey HSD: *P* = .0011). This restricted growth likely reflected the lower nutrient content of pondwater compared to R2A-enriched conditions. For microbial community analysis, bacteria from all four cultivation conditions were harvested for DNA extraction, followed by 16S rDNA amplicon sequencing to generate five replicate sequencing libraries per condition. In addition, raw pondwater (derived from filtered pondwater-sediment slurry) was sampled and sequenced to provide a snapshot of its native bacterial community structure and diversity. Due to low cell counts, three Pondwater_EDEN_ replicates and one raw pondwater replicate failed to yield sufficient DNA for sequencing, resulting in two and four sequencing libraries for these conditions, respectively. The reduced number of Pondwater_EDEN_ replicates limits statistical power so that comparisons against this cultivation condition should be interpreted with caution. In total, 875 882 raw amplicon sequence variants (ASVs) were sequenced from the four cultivation conditions, plus 282 548 from raw pondwater libraries (Supplementary Fig. 3 and 4A). Only sequences appearing at least twice as a unique variant amongst any cultivation condition were classified as ASVs and retained for downstream analysis. To compare community structures between cultivation conditions, unconstrained ordination analysis using Non-metric Multidimensional Scaling (NMDS) with Bray-Curtis distance was performed alongside a permutational multivariate analysis of variance (PERMANOVA), which revealed distinct clustering of microbial communities by cultivation condition (PERMANOVA: *P* = .001, F (4,16) = 2.30; Beta-dispersion: *P* = .85, F (4,16) = 0.33; Fig. 2B). In pairwise comparisons, all groups exhibited significantly distinct centroids (FDR-adjusted *P* < .05) except for Pondwater_EDEN_, which lacked statistical power due to the contribution of only two replicates (Fig. 2B). Notably, whilst R2A_standard_ and R2A_EDEN_ clusters partially overlapped, likely reflecting their common R2A nutrient source, Acclimatised_EDEN_ formed a distinct group, significantly diverging from both the pure R2A-based cultivation conditions. These results showed that Acclimatised_EDEN_ consistently yielded bacterial communities with unique compositions and diversities compared to R2A_standard_ and R2A_EDEN_ cultivation conditions.

To enable comparative analysis across the four cultivation conditions, ASVs were subsampled to the smallest replicate size, yielding 38 852 rarefied sequences within each replicate (Supplementary Fig. 4B). The total number of distinct ASVs was counted, yielding 749 across all replicates. A distinct ASV was defined as a unique sequence variant, one where each ASV was counted only once regardless of how many times it appeared across replicates and cultivation conditions. Strikingly, Acclimatised_EDEN_ yielded significantly more distinct ASVs (333 ± 51, n = 5) than R2A_standard_ (194 ± 73, n = 5), R2A_EDEN_ (123 ± 51, n = 5) and Pondwater_EDEN_ (172 ± 8, n = 2; ANOVA *P* < .0002, F (3, 13) = 13.93; Fig. 2C). These ASVs represented 30 bacterial classes, dominated by Gammaproteobacteria (36.3%), Alphaproteobacteria (22.3%) and Bacteroidia (11.7%). Importantly, Acclimatised_EDEN_ showed the broadest diversity spanning 20 classes, in comparison to just 5, 9 and 14 for R2A_standard_, R2A_EDEN_, and Pondwater_EDEN_, respectively (Fig. 2D). All classes observed in R2A_standard_ and R2A_EDEN_ cultivation conditions were also present in Acclimatised_EDEN_, demonstrating that gradual acclimatisation from native media to R2A using EDEN plates cultivates more diverse bacterial communities than standard methods.

### Acclimatised_EDEN_ polycultures yield the majority of uniquely cultured ASVs

**Figure 3:**
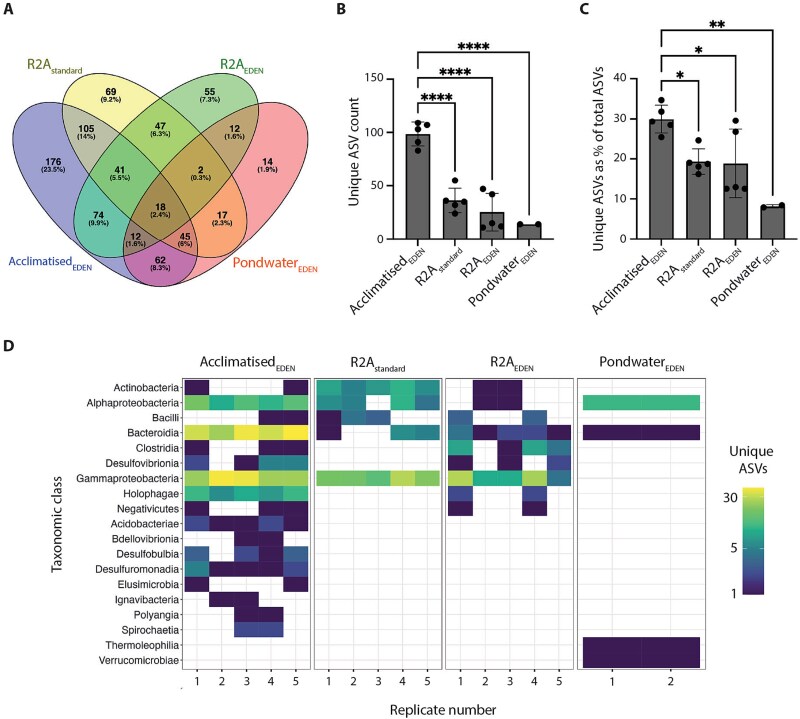
Acclimatised_EDEN_ polycultures yielded the majority of uniquely cultured ASVs. A, Venn diagram showing the proportion of ASVs that were unique to, or shared between cultivation conditions. B, unique ASV count per cultivation condition. Replicates are shown as black dots. Bar height indicates the mean unique ASV count, error bars indicate 1 s standard deviations. C, unique ASV count as a percentage of the total ASVs shown in Fig. 2C. D, heatmap showing the relative abundance and distribution of unique ASVs grouped by taxonomic class. Statistical significance: ^*^P < .05, ^**^P < .01, ^****^P < .0001 (one-way ANOVA and Tukey’s multiple comparison test).

Next, the distribution of distinct ASVs that were shared or grew uniquely across the four cultivation conditions was assessed. Shared ASVs were defined as those ASVs present in at least two cultivation conditions, whilst unique ASVs were present in at least two replicates but only within one cultivation condition. 18 ASVs were common to all cultivation conditions with Gammaproteobacteria (72%) dominating, followed by Alphaproteobacteria (17%) and Bacteroidia (11%; Fig. 3A). In contrast, 314 were unique, cultivated exclusively within Acclimatised_EDEN_, R2A_EDEN_, Pondwater_EDEN_ or R2A_standard_. Strikingly, Acclimatised_EDEN_ accounted for 176 of these unique ASVs—over 2.5-fold more than R2A_standard_, R2A_EDEN_ and Pondwater_EDEN_ with 69, 55 and 14, respectively (Fig. 3A). These unique ASVs were more consistently detected in Acclimatised_EDEN_ replicates (99 ± 11, n = 5) compared to R2A_standard_ (36 ± 11, n = 5), R2A_EDEN_ (25 ± 18, n = 5) and Pondwater_EDEN_ (14 ± 0, n = 2) (ANOVA *P* < .0001 F (3, 13) = 35.18; Fig. 3B). Furthermore, within Acclimatised_EDEN_, unique ASVs accounted for on average 29.9% ± 3.5, n = 5 of the total ASVs in this cultivation condition. This contrasted with 19.3% ± 3.2, n = 5, 18.8% ± 8.6, n = 5, and 8.2% ± 0.4, n = 2, for equivalent comparisons within R2A_standard_, R2A_EDEN_ and Pondwater_EDEN_ (Fig. 3C). Collectively, these findings showed that Acclimatised_EDEN_ yielded significantly more unique ASVs than other cultivation conditions. Within Acclimatised_EDEN_, the majority of unique ASVs belonged to the Burkholderiales (class Gammaproteobacteria, 30.1%), and Bacteroidales (class Bacteroidia, 17.1%; Fig. 3D). A notable proportion of unique ASVs were affiliated with the Holophagae (9.7%) and Acidobacteriotae (0.6%) classes, both members of the underrepresented phylum Acidobacteriota, a group with few cultivated representatives [5]. In comparison, R2A_standard_ mainly produced unique ASVs from the Burkholderiales (26.1%) and Xanthomonadales (class Gammaproteobacteria, 20.3%). Overall, these data showed that Acclimatised_EDEN_ recovers a more diverse and unique set of taxa compared to standard R2A-based cultivation.

### Acclimatised_EDEN_ polycultures yielded significantly increased likely-uncultured ASVs

**Figure 4:**
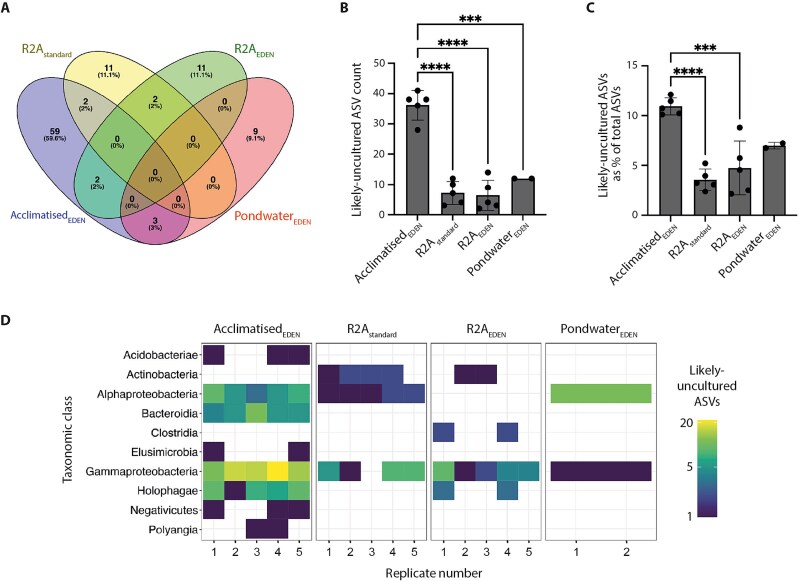
Acclimatised_EDEN_ polycultures yielded significantly increased likely-uncultured ASVs. A, Venn diagram showing the proportion of likely-uncultured ASVs that are either unique to a cultivation condition or shared. B, unique likely-uncultured ASV count per cultivation condition. Replicates are shown as black dots. Bar height indicates the mean unique likely-uncultured ASV count, error bars indicate 1 s standard deviations. C, unique likely-uncultured ASV count as a percentage of the total ASVs shown in Fig. 2C. D, heatmap showing the relative abundance and distribution of unique likely-uncultured ASVs grouped by taxonomic class. Statistical significance: ^***^P < .001, ^****^P < .0001 (one-way ANOVA and Tukey’s multiple comparison test).

Having observed an increase in distinctive diversity in Acclimatised_EDEN_, we examined whether it facilitated growth of bacterial taxa that were likely-uncultured—defined as distinct ASVs lacking genus-level classification in the Silva 16S rRNA gene database [58]. Of the 99 likely-uncultured ASVs identified, few were shared between cultivation conditions (9.1%) whilst most were unique to Acclimatised_EDEN_ (59.6%; Fig. 4A). Shared likely-uncultured ASVs were those ASVs present in at least two cultivation conditions, whilst unique likely-uncultured ASVs were present in at least one replicate in a single cultivation condition. Remarkably, Acclimatised_EDEN_ recovered 59 unique likely-uncultured ASVs, more than four times that obtained from R2A_standard_, R2A_EDEN_ and Pondwater_EDEN_ with 11, 11 and 9, respectively. The recovery of likely-uncultured ASVs across replicates was also consistently higher in Acclimatised_EDEN_ (36 ± 5, n = 5) versus R2A_standard_ (7 ± 4, n = 5), R2A_EDEN_ (6 ± 5, n = 5) and Pondwater_EDEN_ (12 ± 0, n = 2; ANOVA, *P* ≤ .0001, F (3, 13) = 49.28; Fig. 4B). Within Acclimatised_EDEN_, likely-uncultured ASVs represented on average 10.9% ± 0.9, n = 5, of the total ASVs within this cultivation condition (Fig. 4C). This was significantly higher than equivalent comparisons within R2A_standard_ (3.6% ± 1.1, n = 5) and R2A_EDEN_ (4.8% ± 2.7, n = 5). Taxonomic analysis of all likely-uncultured ASVs revealed Acclimatised_EDEN_ to have broad phylogenetic distribution across eight bacterial classes, with particularly high representation of Gammaproteobacteria (44.2%), Bacteroidia (16.6%), and the rarely cultured Holophagae (17.7%; Fig. 4D). In contrast, the other cultivation conditions recovered likely-uncultured ASVs from only 2–4 classes, with Holophagae either absent or poorly represented. Overall, these findings showed that EDEN not only enhanced cultivation of diverse bacteria but was particularly effective at accessing novel microbial taxa that remain likely-uncultured using standard approaches.

### Acclimatised_EDEN_ monocultures yielded increased unique and diverse bacterial taxa

**Figure 5:**
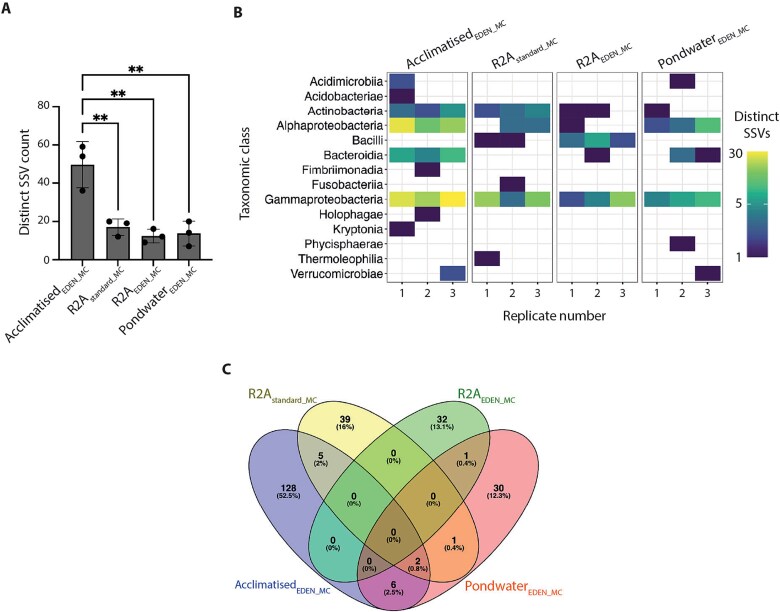
Acclimatised_EDEN_ monocultures significantly increased bacterial abundance and diversity. A, distinct SSV count per cultivation condition. Replicates are shown as black dots. Bar height indicates the mean SSV count, error bars indicate 1 s standard deviations. Statistical significance: ^*^P < .05, ^**^P < .01 (one-way ANOVA and Tukey’s multiple comparison test). B, heatmap showing the relative abundance and distribution of distinct SSVs grouped by taxonomic class. C, Venn diagram showing the proportion of distinct SSVs that are either unique to a cultivation condition or shared.

Having observed enhanced diversity in bacterial polycultures acclimatised using EDEN, we investigated whether similar benefits could be achieved with monocultures (MCs) by inoculating EDEN plates with on average one bacterial cell per well (Poisson λ = 1; Supplementary Fig. 3). The same four cultivation conditions were compared whilst using 384-well EDEN plates—acclimatisation from native pondwater to R2A (Acclimatised_EDEN_MC_) or continuous flow R2A or pondwater (R2A_EDEN_MC_ or Pondwater_EDEN_MC_). For standard cultivation and incubation (R2A_standard_MC_), bacteria from pondwater-sediment slurry were inoculated into R2A within microwell plates. Three independent plate replicates were set up for each cultivation condition. After 28 days, the cultures in each well were harvested and used as templates for 16S rDNA PCR amplification as a measure of cell growth. Acclimatised_EDEN_MC_ yielded the highest mean number of positive wells (155 ± 34, n = 3) though this was not statistically significant compared with R2A_standard_MC_ (71 ± 24, n = 3), R2A_EDEN_MC_ (131 ± 35, n = 3) or Pondwater_EDEN_MC_ (106 ± 129, n = 3) likely due to high variance between Pondwater_EDEN_MC_ replicates (ANOVA *P* = .5313, F (3, 8) = 0.7929; Supplementary Fig. 5). No PCR products were obtained from uninoculated plates (n = 3). To facilitate comparison across cultivation conditions, an equal fraction of PCR products from each cultivation condition were subsampled for Sanger sequencing (Supplementary Fig. 5). Low quality sequences that may derive from mixed cultures were discarded, with the remainder grouped into exact genetic variants (Sanger sequencing variants, SSVs) by assembling them at 100% identity. In this way, identical sequences were grouped together and singletons that did not match other sequences were retained, resulting in 244 distinct SSVs (Supplementary Fig. 5). Notably, Acclimatised_EDEN_MC_ recovered the most distinct SSVs (50 ± 12, n = 3), nearly three-fold higher than R2A_standard_MC_ (17 ± 4, n = 3), R2A_EDEN_MC_ (12 ± 4, n = 3) or Pondwater_EDEN_MC_ (14 ± 7, n = 3) (ANOVA *P* = .0007, F (3, 8) = 17.23; Fig. 5A). Taxonomic analysis showed Acclimatised_EDEN_MC_ supported growth from 10 of the 14 observed bacterial classes, compared to 5–7 classes for other cultivation conditions (Fig. 5B). Subsequent analysis of SSV distribution, either shared or grown uniquely across cultivation conditions, revealed striking differences in microbial recovery. Shared SSVs were defined as those present in at least two cultivation conditions, whilst unique SSVs appeared in only one. Of the total SSVs, 15 were shared across cultivation conditions, whilst 229 were uniquely cultured (Fig. 5C). Acclimatised_EDEN_MC_ outperformed other cultivation conditions, recovering 128 unique SSVs, more than triple the number recovered from R2A_standard_MC_, R2A_EDEN_MC_ and Pondwater_EDEN_MC_ with 39, 32 and 30, respectively. As the number of shared SSVs was relatively low, likely reflecting low sequencing depth relative to the high diversity of pondwater, the unique SSVs accounted for nearly all recovered diversity in each cultivation condition (Supplementary Fig. 6A and B), with taxonomic profiles closely matching those of total SSVs (Supplementary Fig. 6C). Overall, these findings underscored the effectiveness of EDEN to access microbial taxa that may otherwise have remained uncultured with standard methods.

### Acclimatised_EDEN_ monocultures yielded significantly increased likely-uncultured SSVs

**Figure 6:**
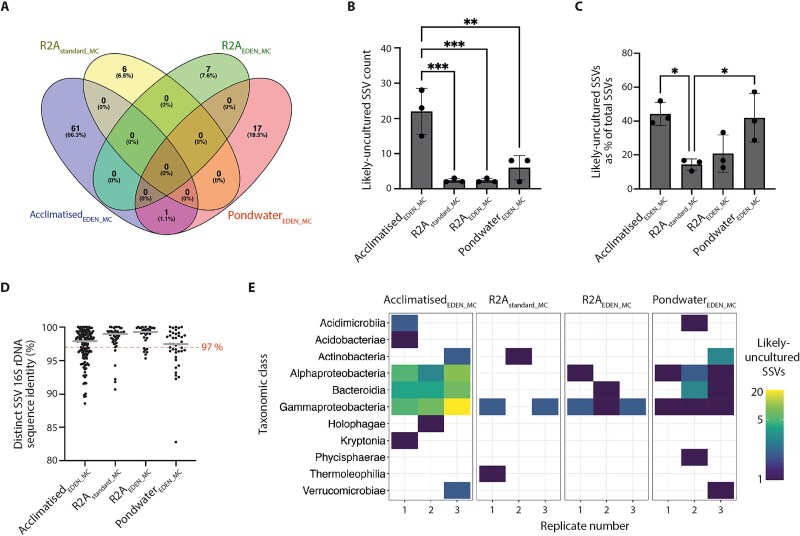
Acclimatised_EDEN_ monocultures yielded significantly increased likely-uncultured SSVs. A, Venn diagram showing the proportion of likely-uncultured SSVs that are either unique to a cultivation condition or shared. B, likely-uncultured SSVs per cultivation condition. Replicates are shown as black dots. Bar height indicates the mean likely-uncultured SSV count, error bars indicate 1 s standard deviations. C, likely-uncultured SSVs as a percentage of the total SSVs shown in Fig. 5A. Pairwise comparisons of Acclimatised_EDEN_MC_ or Pondwater_EDEN_MC_ against R2A_standard_MC_ are statistically significant. D, scatter plot of distinct SSV 16S rDNA sequence identity relative to the SILVA database with median shown in grey lines. E, heatmap showing the relative abundance and distribution of likely-uncultured SSVs grouped by taxonomic class. Statistical significance: ^*^P < .05, ^**^P < .01, ^***^P < .001 (one-way ANOVA and Tukey’s multiple comparison test).

To further assess EDEN’s effectiveness as a cultivation method, we compared the abundance of likely-uncultured SSVs – defined as those distinct SSVs designated “uncultured” in the SILVA database or with <97% 16S rDNA similarity. Remarkably, 67% of likely-uncultured SSVs were recovered from Acclimatised_EDEN_MC_ compared with 6%–17% for the other cultivation conditions (Fig. 6A). For Acclimatised_EDEN_MC_, this translated to a striking 10-fold higher abundance of likely-uncultured SSVs across replicates (22 ± 7, n = 3) compared to R2A_standard_MC_ (2 ± 1, n = 3) and R2A_EDEN_MC_ (2 ± 1, n = 3), and nearly 4-fold more than Pondwater_EDEN_MC_ (6 ± 3, n = 3; ANOVA *P* = .0005. F (3, 8) = 18.98; Fig. 6B). Additionally, Acclimatised_EDEN_MC_ likely-uncultured SSVs accounted for on average 44.2% ± 6.8 (n = 3) of all SSVs in this cultivation condition (Fig. 6C). This contrasted with just 14.3% ± 3.3, n = 3, 20.8% ± 11.0, n = 3, and 41.9% ± 14.4, n = 3, for equivalent comparisons within R2A_standard_MC_, R2A_EDEN_MC_ and Pondwater_EDEN_MC_, respectively (ANOVA *P* = .0127 F (3, 8) = 6.971; Fig. 6C). Moreover, Acclimatised_EDEN_MC_ outperformed other cultivation conditions in growing both likely-cultured or likely-uncultured bacteria (Fig. 6D). Specifically, the median 16S rDNA sequence similarities for Acclimatised_EDEN_MC_ distinct SSVs was 97.9%, compared to R2A_standard_MC_ with 99.0%, R2A_EDEN_MC_ with 99.3%, and Pondwater_EDEN_MC_ with 97.5%. Taxonomic analysis revealed that likely-uncultured SSVs spanned 11 bacterial classes, with Acclimatised_EDEN_MC_ capturing the broadest diversity from 9 of these classes (Fig. 6E). In contrast, R2A_standard_MC_, R2A_EDEN_MC_, and Pondwater_EDEN_MC_ supported growth from 3, 3 and 7 classes, respectively. Furthermore, like polyculture cultivations, Acclimatised_EDEN_MC_ was the only R2A-based cultivation condition to recover SSVs from the underrepresented classes Holophagae, Verrucomicrobiota and Acidimicrobiia, the latter belonging to the phylum Actinomycetota [5, 68] (Fig. 6E). Overall, the relative abundance of Acclimatised_EDEN_MC_ likely-uncultured SSVs, combined with their greater taxonomic diversity, showed that EDEN effectively facilitated access to previously uncultivated taxa.

### EDEN significantly enhanced the cultivability of agarose-encapsulated bacteria

**Figure 7:**
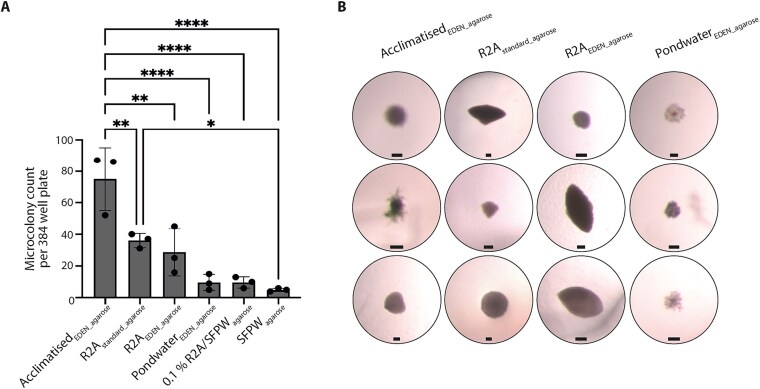
EDEN significantly increases the cultivability of agarose-encapsulated bacteria. A, microcolonies per 384-well plate (n = 3) counted using light microscopy. Statistical significance: ^*^P < .05, ^**^P < .01, ^***^P < .001, ^****^P < .0001 (one-way ANOVA and Tukey’s multiple comparison test). B, gallery of typical microcolony morphologies observed in growth chambers for each cultivation condition. Scale bar = 100 μm.

As microencapsulation combined with *in situ* incubation has previously improved microbial recovery (28, 55), we tested whether EDEN could promote the cultivability of agarose-encapsulated bacteria. Bacteria from pondwater-sediment slurry were mixed with low-gelling-point agarose and inoculated into 384-well EDEN plates with on average one cell per well (Poisson λ = 1). The same cultivation conditions were tested as before with acclimatisation from native pondwater to R2A (Acclimatised_EDEN_agarose_) or continuous flow R2A (R2A_EDEN_agarose_) or pondwater (Pondwater_EDEN_agarose_). For comparison, the same bacterial sample was mixed with R2A agarose within microwell plates for standard static cultivation (R2A_standard_agarose_). As an additional comparison, bacteria were mixed with sterile-filtered pondwater agarose ±0.1% R2A and dispensed into 384-well microwell plates. Three independent plate replicates were set up for all cultivation conditions. After 28 days, microcolonies in each EDEN or microwell plate were counted by light microscopy. Microcolonies were clearly distinguishable from uninoculated control wells. Acclimatised_EDEN_agarose_ produced over twice the number of microcolonies (75 ± 41, n = 3) compared with R2A_standard_agarose_ (36 ± 18, n = 3) and R2A_EDEN_agarose_ (29 ± 19, n = 3; ANOVA F (5, 12) = 18.46, *P* < .001; Fig. 7A and B). Additionally, significantly fewer microcolonies were counted in Pondwater_EDEN_agarose_ (10 ± 6, n = 3) and in the microwell plates containing sterile-filtered pondwater agarose (5 ± 3, n = 3; or 10 ± 6, n = 3 when supplemented with 0.1% R2A). Sterile-filtered pondwater agarose conditions therefore yielded the fewest colonies, consistent with previous observations where pondwater supported minimal growth (Fig. 2A). Overall, these results showed that gradual acclimatisation from native media to R2A using EDEN plates significantly improved microcolony formation.

### EDEN-cultivated isolates exhibited broad-spectrum antimicrobial activity

**Figure 8:**
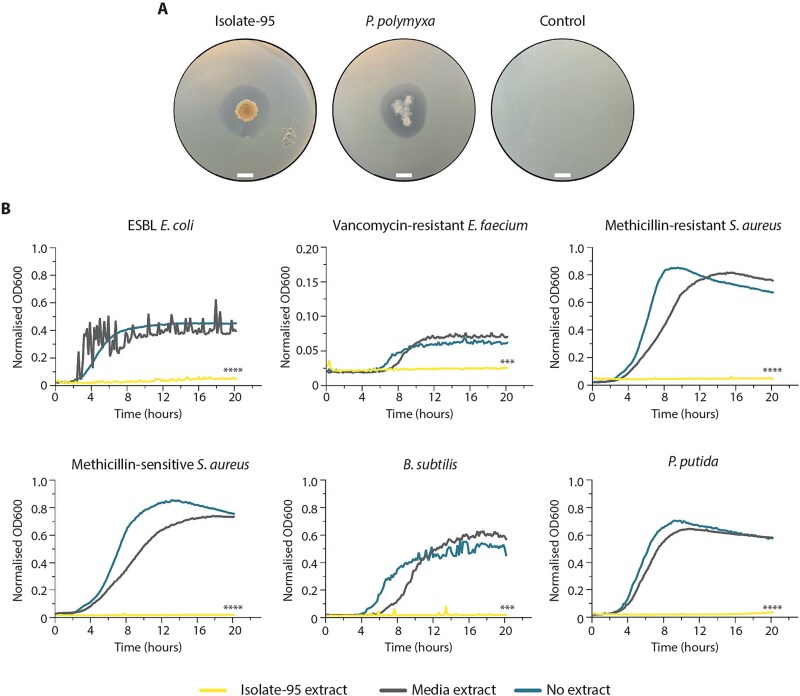
An EDEN cultivated isolate exhibits potent broad-spectrum antimicrobial activity. A, growth-inhibition assay with Isolate-95 and with known antibiotic-producing *Paenibacillus polymyxa* grown in the petri dish overlaid with MRSA lawn. Antimicrobial activity was detected as a clearance zone around the central colony. Scale bar = 1 cm. B, liquid culture growth-inhibition assays with extended spectrum beta lactamase (ESBL) *E. coli*, vancomycin resistant *Enterococcus faecium*, methicillin resistant and methicillin sensitive *Staphylococcus aureus* (MRSA and MSSA), *B. subtilis*, and *Pseudomonas putida* grown in the presence of crude Isolate-95 extract (yellow), sterile media extract (grey), or without extract (blue). Isolate-95 extract significantly inhibited the growth of all tested bacteria, whilst no growth inhibition was observed using sterile media extract or with no extract (average of n = 3 independent replicates). Statistical significance was tested against the negative control (no extract) on endpoint growth measurements: ^***^P < .001, ^****^P < .0001 (one-way ANOVA and Dunnett’s multiple comparisons test).

Given that previously uncultivated bacteria represent abundant sources of novel bioactive compounds [9, 69], we screened isolates from Acclimatised_EDEN_ polycultures for antimicrobial activity using growth inhibition assays against methicillin-resistant *Staphylococcus aureus* (MRSA). Remarkably, among just 400 colonies tested where 367 (91.8%) were successfully subcultivated on R2A agar, one isolate, termed Isolate-95, exhibited potent activity comparable to the known antimicrobial producer *Paenibacillus polymyxa* (Fig. 8A). Genome BLAST Distance Phylogeny (GBDP) analysis placed the isolate on a distinct phylogenetic branch within the Alphaproteobacteria, clearly separated from its closest relatives, including *Rhodobacter tardus* (19.9% digital DNA–DNA hybridization, dDDH, formula d4; Supplementary Fig. 7A). Consistent with this placement, average amino acid identity (AAI) comparison with the Microbial Genomes Atlas (MiGA [66]) revealed low similarity to the nearest reference species *Cypionkella sinensis (*AAI = 62.3%). Statistical support indicated the isolate represented a previously undescribed species (*P* = .0056) and possibly a novel genus (*P* = .43). Neither species were previously reported to exhibit antimicrobial activity.

Genome analysis of Isolate-95 identified 6 predicted BGCs (Supplementary Fig. 7B), all of which appeared novel except for the carotenoid terpene-type cluster that may produce the dark red colony pigmentation (Fig. 8A). Notably, none showed similarity to antimicrobial-associated BGCs in the MIBiG 3.1 database [70]. This included the RiPP-like cluster, which belongs to a diverse family of bioactive compounds encompassing antimicrobials [71]. To test for broad spectrum antimicrobial activity, we cultured Isolate-95 on TY agar before using ethyl acetate to generate a crude extract. Control extracts were made using sterile TY agar (Fig. 8B). Notably, Isolate-95 extract exhibited strong growth inhibition against multiple drug-resistant pathogens including extended-spectrum beta-lactamase (ESBL) resistant *E. coli* (88.3% ± 4.3, n = 3, Dunnett’s test: *P* < .0001), vancomycin-resistant *Enterococcus faecium* (60.3% ± 5.5, n = 3, Dunnett’s test: *P* = .0005) and methicillin-sensitive *Staphylococcus aureus* (MSSA; 92.4% ± 7.0, n = 3, Dunnett’s test: *P* < .0001). Additionally, the extract effectively inhibited environmental strains of *Pseudomonas putida* (93.5% ± 1.5, n = 3, Dunnett’s test: *P* < .0001), MRSA (97.3% ± 0.3, n = 3, Dunnett’s test: *P* < .0001) and *B. subtilis* (95.8% ± 0.4, n = 3, Dunnett’s test: *P* = .0004). These findings established the broad-spectrum antimicrobial activity of Isolate-95, which may originate from the novel BGCs identified or from other undetected clusters.

## Discussion

We present EDEN, a method for the enhanced domestication of microorganisms that gradually acclimatises bacteria to culture media using a 3D-printed continuous-flow cultivation plate. The method improves taxonomic diversity, yields more unique and likely-uncultured taxa and increases microcolony recovery. Using EDEN, we isolate a novel bacterium with antimicrobial activity against multiple drug-resistant pathogens.

Whilst the adaptive mechanism by which EDEN increases bacterial cultivation and diversity remains unclear, it may include a complex interplay of epigenetic [72], physiological, and metabolic factors [73–76], or resuscitation from dormancy [77]. Notably, epigenetic phase variation driven by DNA methylation may regulate gene expression and promote phenotypic heterogeneity and viable subpopulation growth in clonal populations. Examples of this include the rapid adaptation of *Ralstonia pseudosolanacearum* to novel plant host-related environments [78] or facilitating adaptation within the human microbiome [79]. Alternatively, genetic mutation may enhance fitness and cell growth [80], occurring as a response to cell stress triggered by poor adaptation to available nutrients [81].

There are various established and innovative techniques for increasing the cultivability and domestication success of microorganisms [5, 28–31]. EDEN has several features that distinguishes itself from such methods, whilst also presenting numerous advantages over standard cultivation methods where microorganisms are inoculated and grown in liquid or solid substrate culture media. By initiating bacterial domestication using pondwater as a native media, EDEN purports to mimic the native environment. This minimises bacterial exposure to abrupt environmental changes [28, 30] that may compromise the viability of sensitive taxa [39, 41, 82] and hinder their potential for adaptation. The native media may also impede fast growing bacteria, inherently adapted to nutrient-rich target culture media, from outcompeting oligotrophs or slower growing taxa. Such an effect is evidenced by R2A_EDEN_ yielding a significantly different community structure to Acclimatised_EDEN_ (Fig. 2B) with substantially fewer distinct ASVs and less diversity (Fig. 2C and D) despite cultivating approximately four times higher cell numbers (Fig. 2A). Finally, the native media retains microbes from the source community that may initially provide essential growth stimuli through cell–cell signalling or co-cultivation symbioses before becoming dispensable [21].

EDEN implements a gradual transition to desired culture media so that uncultured bacteria may persist long enough to adapt to the changing environment. This strategy supports uncultured bacterial taxa to navigate multifaceted adaptive landscapes that may ultimately result in their domestication [80]. Of course, bacteria have defined genetic, physiological, and metabolic capacities whose plasticity limits their adaptability so that only a subset of taxa have the potential to thrive within any given culture medium. To capture a broader taxonomic range, EDEN plates acclimatising into different culture media other than R2A would likely be effective, as evidenced by the distinct communities and diversity profiles grown between Acclimatised_EDEN_, R2A_EDEN_ and Pondwater_EDEN_ conditions (Figs 2B–D), with a similar effect observed between monocultures (Fig. 5B and C). Further expansion of cultivated taxa may be achieved by using different acclimatisation protocols beyond the exponential gradient tested here (Fig. 1A). Potential alternatives include linear gradients or stepped approaches incorporating staged media concentration spikes over variable timelines.

By gradually shifting from native to culture media, EDEN achieves significantly greater taxonomic diversity, and cultivates more unique and likely-uncultured taxa. This improvement was evident by comparing Acclimatised_EDEN_ against the less effective cultivation performance of R2A_EDEN_ and Pondwater_EDEN_ where continuously flowing R2A or pondwater were exclusively used as nutrient sources (Figs 2–4). The same effect was observed for equivalent monocultures (Fig. 5 and 6). Moreover, Acclimatised_EDEN_ outperformed R2A_standard_ cultivation conditions in all tested formats—a notable outcome given R2A was specifically optimised for microbial cultivation from aqueous environments [50]. In addition, as Acclimatised_EDEN_ culminates its transition in pure culture media conditions, any reliance on native growth factors for cultivation may be diminished, potentially avoiding future requirement to return to, or emulate, native environments for long-term domestication [26]. In this respect, serial subcultivation was attempted for just 400 colonies isolated from Acclimatised_EDEN_ polycultures. Whilst 91.8% of these colonies subcultivated, further studies are required to comprehensively characterize the long-term domestication outcomes derived from EDEN acclimatisation across all experimental formats.

In addition to bacteria, EDEN may be effective for the cultivation of some fungi [83] or archaea [49], many of which remain uncultured. Alternatively, the EDEN plate may be applied to developing tolerance against antagonistic substances [84, 85], used as a perfusion bioreactor [86], or combined with microbial whole cell biosensors with diverse applications [87]. The use of 3D-printing and print-pause-print technique to fabricate EDEN plates eliminated the need for manual assembly or adhesives with dubious biocompatibility [88]. Furthermore, it supports future development of the EDEN plate with further innovations including additional layers of growth or reporter chambers, or media reservoirs, separated by membranes. The standard microwell format of the EDEN plate integrates with liquid handling robots, enabling high-throughput screening of novel microbial diversity for beneficial natural products beyond antimicrobials [11, 89, 90].

Overall, EDEN constitutes an innovative and effective cultivation method that successfully bridges the transition between natural habitats and laboratory conditions. The method demonstrates substantial improvements in microbial recovery, taxonomic diversity, and access to novel taxa compared with standard cultivation techniques. The vast uncultured majority remains an important source of novel natural products including antimicrobials, as demonstrated by the discoveries of Teixobactin and Malacidin [9, 91]. By culturing novel bacteria, EDEN may prove similarly useful for discovering uncharted bioactive compounds.

## Supplementary Material

ycag062_Supplemental_Files

## Data Availability

All raw 16S rDNA amplicon sequencing data generated in this study are available in the NCBI Sequence Read Archive (SRA) under BioProject PRJNA1299469. Sanger sequencing data for cultured SSVs are available in GenBank under accession numbers PX068647-PX068882. ASV abundance table, taxonomic classification and raw data for statistical analyses are presented in Supplementary data. 3D model files for EDEN plates and code for Arduinos and 3D printers are available at Figshare (DOI: 10.6084/m9.figshare.c.7989857).
