# Supplementary material for: An enhanced domestication method for uncultured bacteria
Source: ISME Commun. 2026 Mar 16;6(1):ycag062. doi: 10.1093/ismeco/ycag062 (PMC13082232; doi:10.1093/ismeco/ycag062)
Supplement: ycag062_Supplemental_Files [file ycag062_supplemental_files.zip › Morrison_Low_SI_revised_ycag062.pdf]

An enhanced domestication method for uncultured bacteria

Supplementary Information

Andrew G. Morrison[1], Raphaella Jackson[1,2], Paul S. Freemont[1], Harry H. Low[1]*

Affiliations:

[1]Department of Infectious Disease, Imperial, London, UK

[2]Current address: Department of Women and Children's Health, King's College, London, UK

*Corresponding author: h.low@imperial.ac.uk

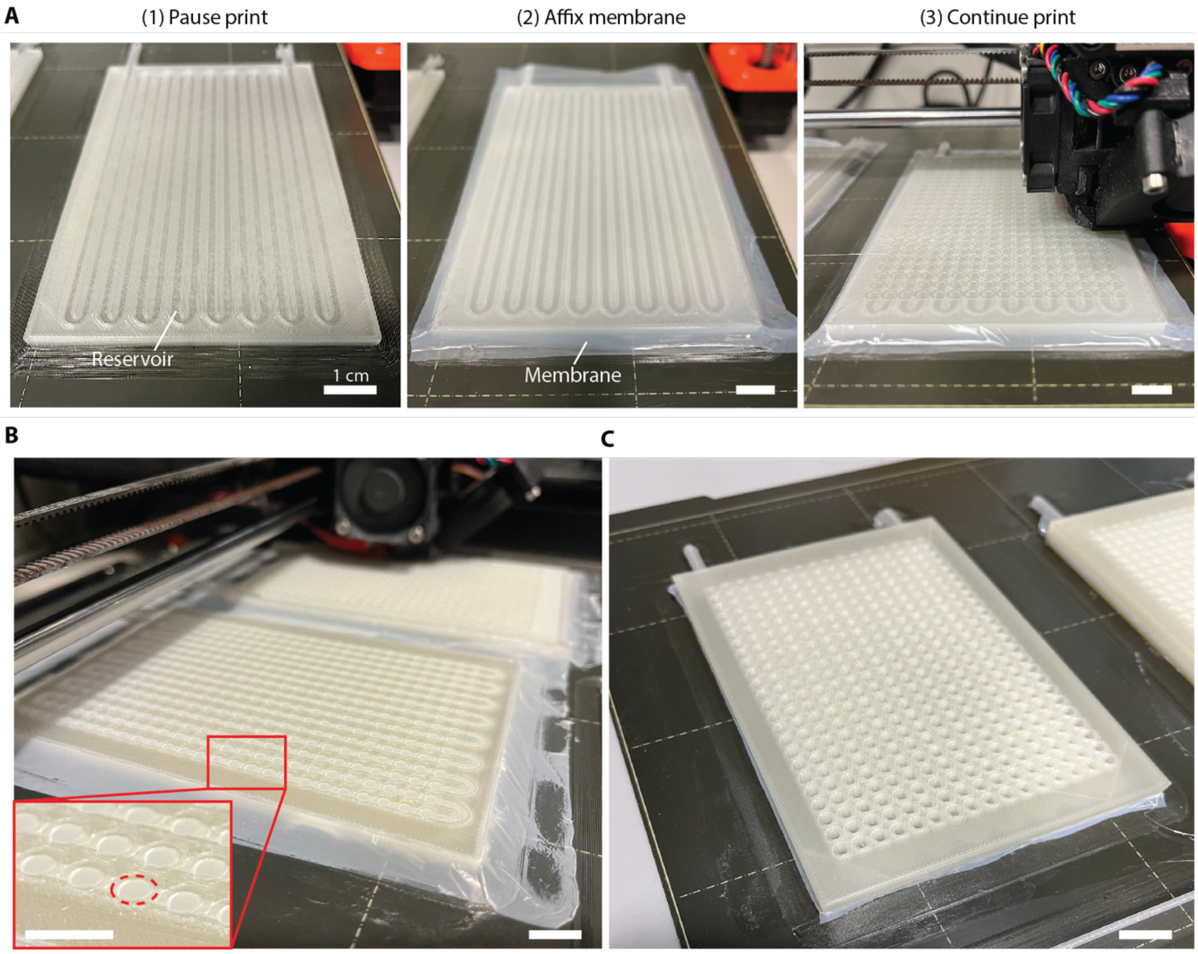

Supplementary Figure 1. EDEN plate 3D print fabrication with integrated polycarbonate membrane using print-pause-print technique. A, Step 1: the reservoir is 3D printed with automatic pausing of the print before starting the growth chambers. (2) Polycarbonate membrane is placed over the printed reservoir and secured to the printer bed with PVA glue. (3) The first layer of the growth chambers is printed and heat-bonded to the membrane thereby removing the requirement for external adhesive. **B,** Printing of 384-well growth chambers. Zoom box shows detail of partially printed growth chambers with the first lay printed over the membrane. Dashed red circle indicates the well boundary. Growth chambers and the reservoir are connected by the semi-permeable membrane. **C,** Two completed EDEN plates 3D printed simultaneously. Scale bar = 1 cm.

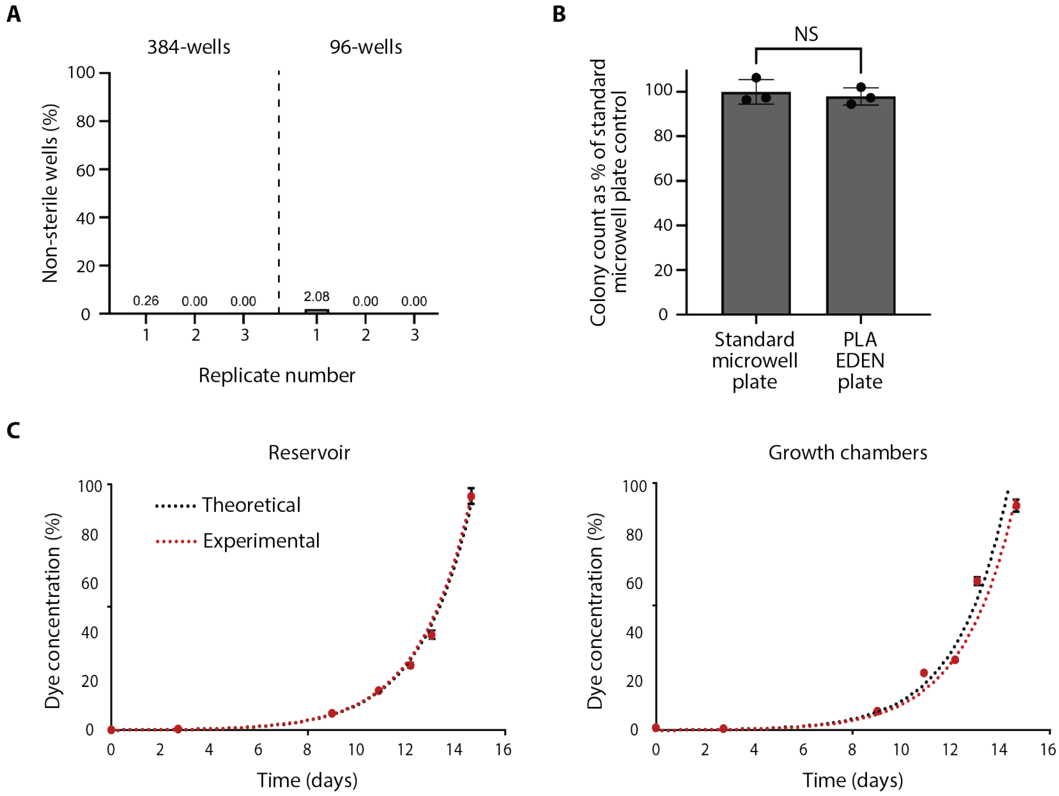

Supplementary Figure 2. Validation of EDEN plate integrity and pump system accuracy. A, Membrane integrity testing after EDEN plate fabrication. *P. putida* leakage from the reservoir to the growth chamber occurred in 1/1152 wells (384-well plates) and 2/288 wells (96-well plates). **B,** EDEN plate polylactic acid (PLA) biocompatibility assessment. *B. subtilis* colony counts on R2A agarose in sterilised EDEN plates were quantified as a percentage of colonies cultivated on standard microwell plates. NS, not significant (unpaired t-test). **C,** The peristaltic pump system accurately generated an exponential gradient of fluorescein fluorescent dye within the EDEN plate. The experimental gradient (black dots) closely matched theory (red dots) in both the continuous-flow reservoir (left) and growth chambers (right) over two weeks. Time points represent mean ± 1σ, n=3 independent replicates.

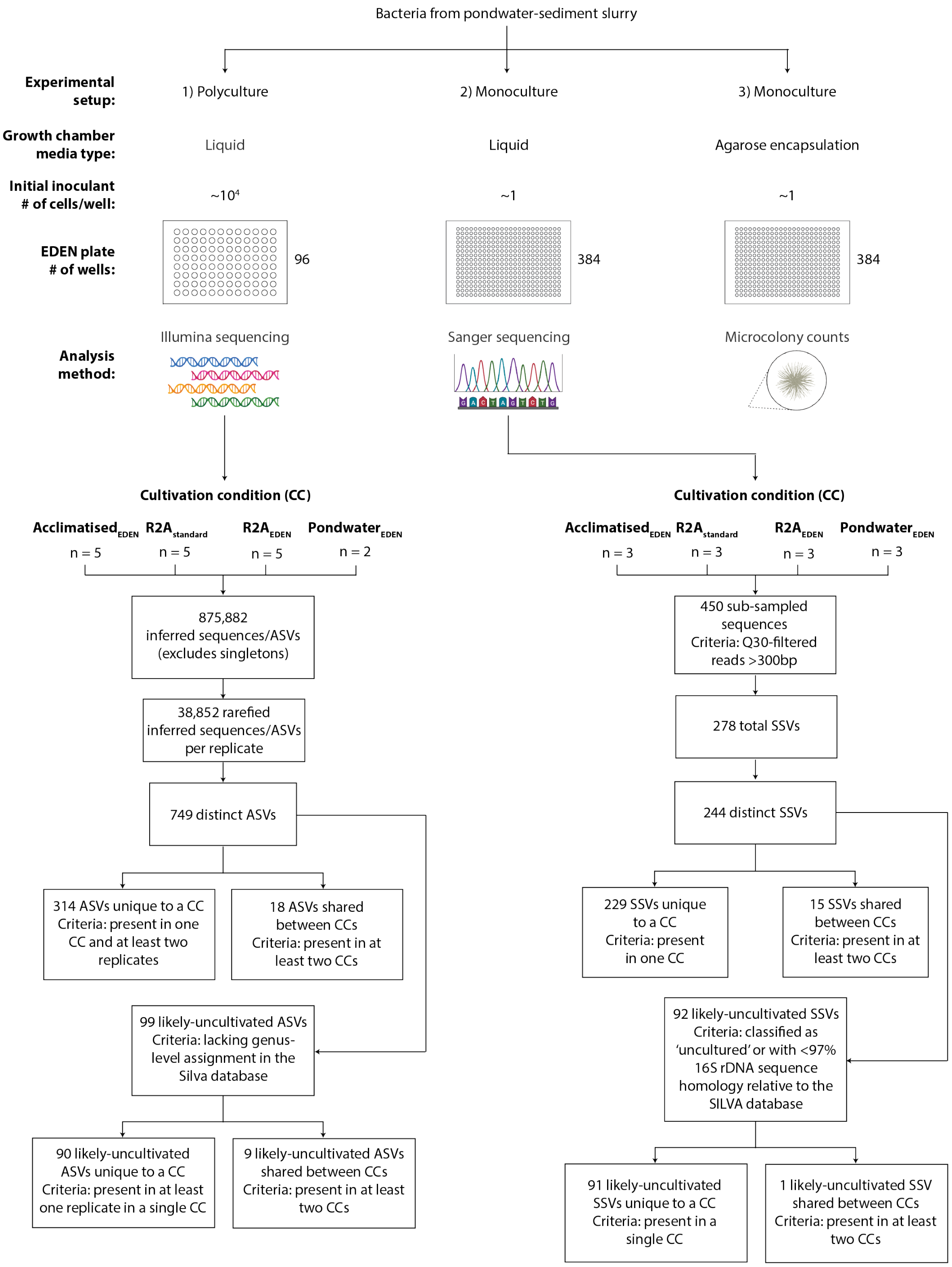

Supplementary Figure 3. Experimental workflow with flowcharts illustrating processing and analysis pipelines for Illumina and Sanger sequencing data. Three experimental setups were implemented each incorporating four cultivation conditions (CC): Acclimatised$_{EDEN}$, R2A$_{EDEN}$, Pondwater$_{EDEN}$ and R2A$_{standard}$.

A

	Raw sequences (Cutadapt input)	DADA2 input	Quality filtering	Denoised forward	Denoised reverse	Successfully merged pairs	Non-chimeric sequences	Inferred bacterial sequences/ASVs[1]
AcclimatisedEDEN1	61621	60847	51325	51162	51277	50252	48730	48730
AcclimatisedEDEN2	50647	49996	41278	41185	41166	40237	38852	38851
AcclimatisedEDEN3	83707	82708	70277	70190	70229	69633	69276	69272
AcclimatisedEDEN4	73219	72309	60349	60223	60322	59683	58767	58767
AcclimatisedEDEN5	76454	75511	63747	63692	63741	63452	62818	62818
Raw pondwater1	89277	88261	76602	74524	75330	69310	68292	68258
Raw pondwater2	70257	69403	59492	57648	58546	53267	52370	52349
Raw pondwater3	75364	74334	63743	62119	62748	57675	56821	56781
Raw pondwater4	68146	67418	55854	54111	54703	49202	48461	48416
PondwaterEDEN1	66446	65619	56841	56803	56836	56744	56744	56744
PondwaterEDEN2	67737	66905	58865	58829	58844	58641	58638	58638
R2AEDEN1	66177	65408	57220	56942	57170	55644	54380	54380
R2AEDEN2	71866	71077	62322	62134	62255	60543	58297	58297
R2AEDEN3	74799	73940	64893	64608	64800	63697	59909	59909
R2AEDEN4	68406	67702	59114	58877	59052	57348	55134	55134
R2AEDEN5	57582	56903	49227	49027	49151	48274	46820	46820
R2Astandard1	69009	68185	58091	57753	57986	56405	54997	54997
R2Astandard2	61963	61193	51510	51186	51431	50510	47760	47760
R2Astandard3	79360	78371	65214	64980	65107	64367	61840	61840
R2Astandard4	60407	59679	51315	50782	51180	49742	48995	48995
R2Astandard5	64188	63428	53665	53256	53594	52205	50674	50674

[1]After removing sequences assigned to mitochondria, chloroplasts or unclassified domains.

B

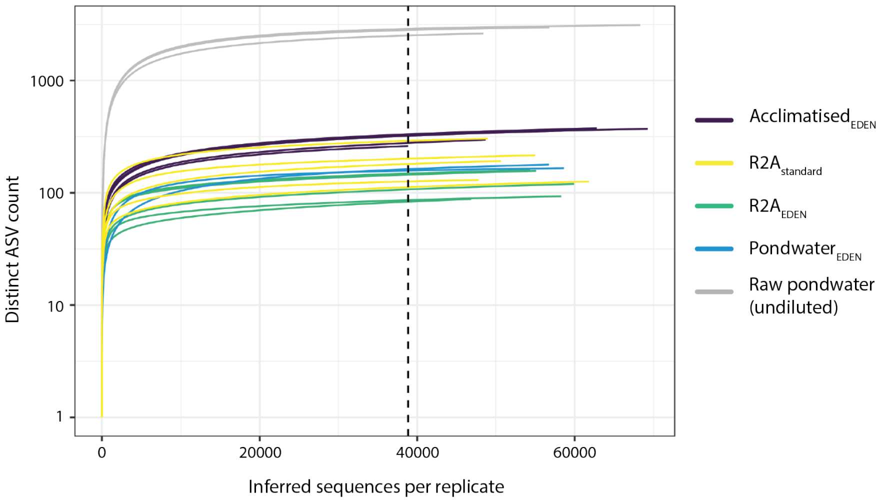

Supplementary Figure 4. Illumina MiSeq sequence data and ASV rarefaction curve. A, Summary of Illumina sequence processing using the Ampliseq pipeline which includes Cutadapt and DADA2 for read/sequence trimming, quality filtering, denoising, chimera removal and ASV inferral. **B**, Rarefaction curve illustrating the cumulative number of distinct ASVs as a function of sequencing depth. Dashed line indicates sequence subsample threshold (38,852).

Cultivation condition	PCR products (from 384 reactions)	Sanger sequenced[1]	Q30 filtered >300bp reads	Total SSVs[2]	SSVs per cultivation condition[3]	Distinct SSVs[4]
AcclimatisedEDEN1	183	69	69	59		
AcclimatisedEDEN2	118	44	44	36	141	
AcclimatisedEDEN3	165	62	62	54		
R2Astandard1	99	37	37	19		
R2Astandard2	57	21	18	12	47	
R2Astandard3	58	22	22	20		
R2AEDEN1	92	35	35	9		
R2AEDEN2	144	54	54	12	33	
R2AEDEN3	158	60	60	16		
PondwaterEDEN1	18	7	7	7		
PondwaterEDEN2	45	17	17	14	40	
PondwaterEDEN3	254	96	25	20		
Total	1391	524	450	278	261	244

[1] Based on an equal proportion of subsampled PCR products.
[2] Includes duplicate SSVs between cultivation conditions and replicates, but not within individual replicates. Dataset for statistical analysis.
[3] Like [2] but excludes duplicate SSVs between replicates within cultivation conditions.
[4] Distinct SSVs, which excludes duplicate SSVs between cultivation conditions, as presented in Venn diagrams and used for unique/shared classifications.

Supplementary Figure 5. Sanger sequencing and SSV data summary. PCR products from each replicate were proportionally subsampled for Sanger sequencing. High quality Q30 filtered >300 bp reads were clustered into SSVs. The PCR detection limit was 5370 ± 420 (n=3) and 6450 ± 530 (n=3) colony forming units (CFUs) per well using *B. subtilis* and *P. putida* as controls, respectively.

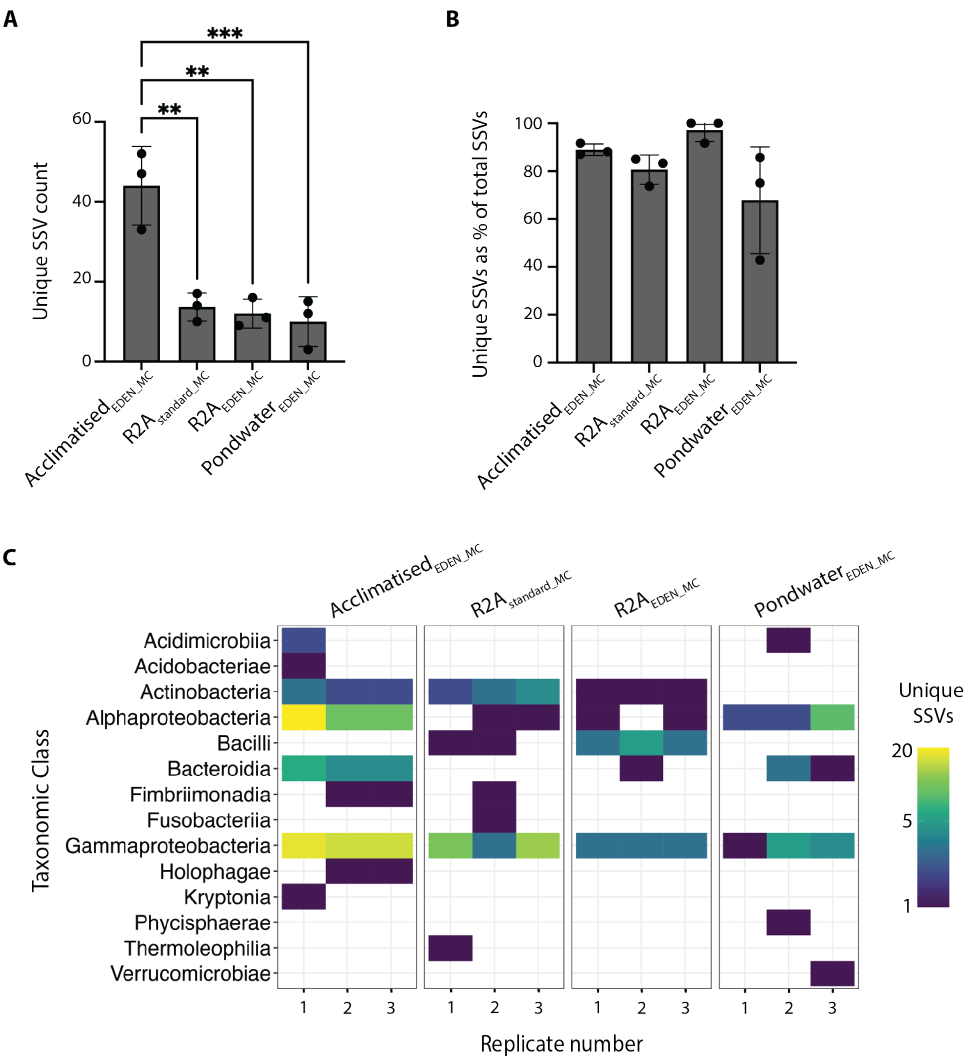

Supplementary Figure 6. Acclimatised$_{EDEN}$ monocultures produced significantly higher unique SSV counts and enhanced bacterial diversity. A, Unique SSV count per cultivation condition. Replicates are shown as black dots. Bar height indicates the mean unique SSV count, error bars indicate 1σ standard deviations. Acclimatised$_{EDEN_MC}$ was significantly higher (44 ± 10, n=3) than R2A$_{standard_MC}$ (14 ± 4, n=3), R2A$_{EDEN}$ (12 ± 4, n=3) and Pondwater$_{EDEN_MC}$ (10 ± 6, n=3; ANOVA p = 0.0005, F (3, 8) = 19.34). **B**, Unique SSVs as a percentage of the total SSVs shown in Fig. 5A. As shared SSV count was relatively low (Fig. 5C), the unique SSVs accounted for nearly all the SSVs in each cultivation condition. **C**, Heatmap showing the relative abundance and distribution of unique SSVs grouped by taxonomic class. Statistical significance: ** p < 0.01, *** p < 0.001 (one-way ANOVA and Tukey's multiple comparison test).

A

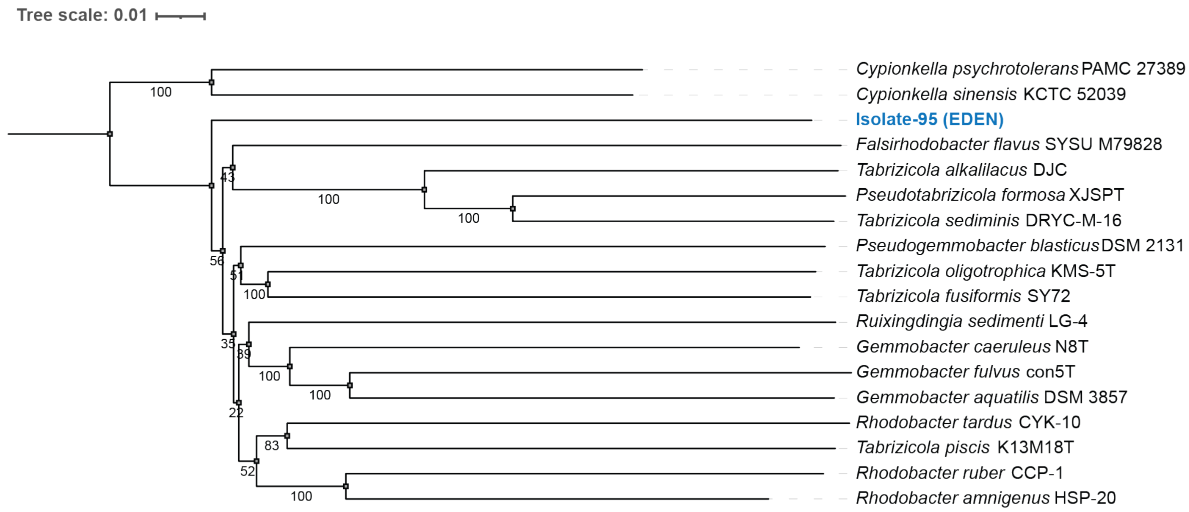

B

Detected genomic BGC	BGC type	Genome BGC start (bp)	Genome BGC end (bp)	Similar known cluster	Similarity confidence
Region 1	Hydrogen-cyanide	526838	547164	N/A	N/A
Region 2	Terpene-precursor	897736	918602	N/A	N/A
Region 3	RiPP-like	1509461	1520336	N/A	N/A
Region 4	Terpene	2674445	2706015	Carotenoid	High
Region 5	Homoserine lactone	3883794	3904498	N/A	N/A
Region 6	Hydrogen-cyanide	4571998	4584743	N/A	N/A

Supplementary Figure 7. Genomic and phylogenetic analysis of Isolate-95 isolated using EDEN. A, Isolate-95 phylogenetic tree. The branch lengths are scaled in terms of Genome BLAST Distance Phylogeny (GBDP) distance formula d5. The numbers above branches are GBDP pseudo-bootstrap support values > 60 % from 100 replications, with an average branch support of 72.1 %. The tree was rooted at the midpoint. **B**, Predicted BGCs based on antiSMASH whole genome analysis. BGCs included a ribosomally-synthesised and post-translationally modified peptide-like (RiPP-like) type that often code for antimicrobials, two hydrogen cyanide types, one terpene-precursor and terpene, and one homoserine lactone. Volatile compounds such as hydrogen-cyanide likely evaporate during extraction and thus are unlikely to contribute to observed antimicrobial activity.
